# A case of relapsing visceral leishmaniasis: the potential role of steroids and RANKL inhibitors in worsening disease outcome

**DOI:** 10.1128/asmcr.00006-25

**Published:** 2025-05-08

**Authors:** Irene Zaghi, Margherita Ortalli, Alessandra Mistral De Pascali, Bianca Granozzi, Alessandra Cascavilla, Luciano Attard, Alessandro Deni, Stefania Varani

**Affiliations:** 1 Department of Medical and Surgical Sciences, University of Bologna 9296, Bologna, Italy; 2 Unit of Microbiology, IRCCS Azienda Ospedaliero-Universitaria di Bologna, Bologna, Italy; 3 Infectious Diseases Unit, IRCCS Azienda Ospedaliero-Universitaria di Bologna, Bologna, Italy; Vanderbilt University Medical Center, Nashville, Tennessee, USA

**Keywords:** visceral leishmaniasis, *Leishmania infantum*, immunocompromised patient, denosumab, monitoring

## Abstract

**Background:**

Individuals pharmacologically immunosuppressed, whether by chemical or biological drugs, are prone to severe infections, including leishmaniasis. In *Leishmania*-infected immunocompromised patients, numerous factors contribute to the clinical picture and response to treatment, including the parasite, the immune system, and the co-medications.

**Case Summary:**

Here, we present the case of a 2-year relapsing visceral leishmaniasis (VL) in an immunocompromised patient suffering from undifferentiated connective tissue disease and undergoing treatment for osteoporosis with a monoclonal antibody targeting the bone resorption mediator receptor activator of nuclear factor-κB ligand (RANKL). The ultimate resolution of VL was achieved by an integrated approach that included the administration of combined antiparasitic treatment followed by secondary prophylaxis, tapering of immunosuppression including drastic reduction of oral steroids and suspension of hydroxychloroquine and the suspension of RANKL inhibitor. As RANKL restrains *Leishmania* replication by activating macrophages, we hypothesize that the RANKL inhibitor may have synergized with the immunosuppressive treatment in inducing parasite expansion and relapsing of VL in the index case.

**Conclusion:**

This case highlights the need to consider *Leishmania*-infected immunocompromised patients as complex systems; numerous factors can be implicated in the balance between the parasite, the immune system, and co-medications and may contribute to the response to treatment.

## INTRODUCTION

Visceral leishmaniasis (VL) is a parasitic disease that is emerging in northern Italy (1). In immunocompromised patients, VL is characterized by high rates of treatment failure and relapses (2). Nevertheless, little is known about the molecular mechanisms behind resistance to treatment, how immunosuppression determines parasite persistence, and the most appropriate treatment regimen in different groups of patients with impairment of the immune response. We present the case of an immunocompromised patient suffering from relapsing VL with a highlight on antiparasitic treatment and management of immunosuppression.

## CASE PRESENTATION

In September 2018, a 61-year-old woman presented with long-term fever and malaise at the Infectious Disease Unit, University Hospital of Bologna, (Emilia-Romagna, northeastern Italy). On admission, blood count showed pancytopenia (white blood cells 2.200/mmc, [neutrophils 1.610, lymphocytes 400], hemoglobin 9.3 g/dL, platelets 56.000/mmc) and abdominal imaging revealed splenomegaly. The patient was suffering from undifferentiated connective tissue disease, for which she was receiving oral steroids (methylprednisolone 16 mg per day) and hydroxychloroquine (200 mg per day); she had recently stopped methotrexate, which she had taken for several years before this episode. Because of severe osteoporosis induced by steroids, in July 2018 she started treatment with denosumab, a human monoclonal antibody targeting the key bone resorption mediator RANKL, which is administered subcutaneously once every 6 months and prevents bone remodeling (3).

**FIG 1:**
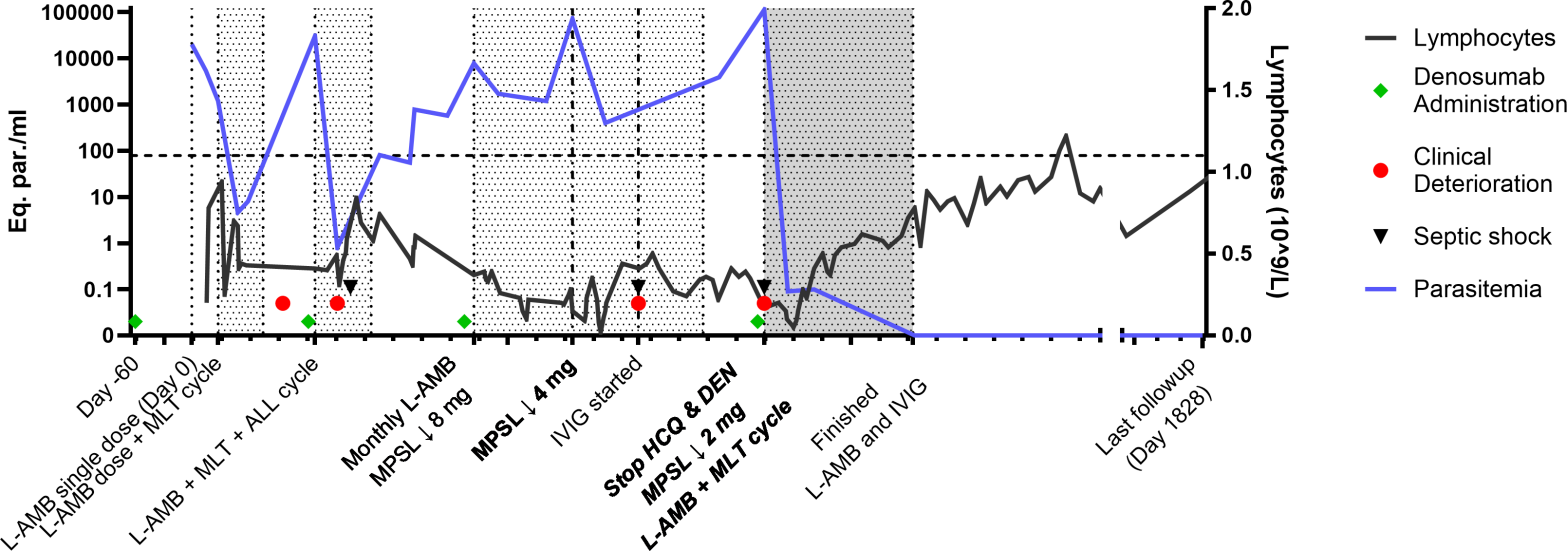
Course of relapsing visceral leishmaniasis (VL) in the index patient. Representation of detailed disease course in the index patient, including parasitemia timeline, antiparasitic and immunological treatments (described on the X-axis), clinical events, and lymphocyte count. An ultrasensitive real-time PCR was carried out in peripheral blood samples to amplify the kinetoplast DNA (kDNA) as described (5). A standard curve was set up for the detection of kDNA, and parasitemia was measured (blue line; numbers depict the parasite levels expressed as parasite equivalents/mL). Denosumab (green diamonds) was administered at a dosage of 60 mg every 6 months and discontinued in May 2020, as well as hydroxychloroquine. Black triangles depict episodes of septic shock. Red circles represent VL relapses identified as worsening of pancytopenia and fever that required hospital admission. X-axis: each small tick depicts 1-month interval. The treatment cycles are described in detail in the case presentation. The black line represents the lymphocyte count; lymphocyte count and kDNA PCR were performed in parallel; only selected time points for lymphocyte count are depicted in the graph. The horizontal dotted line depicts the lower range of normal lymphocyte count. The shaded areas represent antiparasitic treatment cycles. Monthly L-AMB (3 mg/kg) was used from month 10th to month 18th.

Because of the local re-emergence of human leishmaniasis (4), VL was promptly suspected after hospital admission; the patient tested positive for anti-*Leishmania* IgG by enzyme immunoassay and VL diagnosis was confirmed by a positive result in peripheral blood by real-time PCR (Fig. 1). The patient was treated with a single dose of 10 mg/kg liposomal amphotericin B (L-AMB) with resolution of symptoms after 6 days and partial recovery of the pancytopenia.

After this first partial resolution of clinical VL, the patient suffered several episodes of malaise and fever accompanied by severe pancytopenia and persistent positive *Leishmania* DNA in peripheral blood, for which she received the following treatments (Fig. 1); (i) single dose of L-AMB 10 mg/kg followed by miltefosine 50 mg three times a day for 28 days; (ii) combined treatment including L-AMB 4 mg/kg per day for 5 days followed by L-AMB 3 mg/kg once a week for 5 weeks, miltefosine 50 mg three times a day for 28 days, and allopurinol 300 mg twice a day for 2 weeks; (iii) L-AMB 3 mg/kg once a month for 8 months; and (iv) simultaneous administration of L-AMB 4 mg/kg per day for 5 days and miltefosine 50 mg three times a day for 28 days, followed by L-AMB 4 mg/kg once a month for 6 months.

Parasitemia was monitored by employing an in-house quantitative (q)PCR (Fig. 1); despite a slow but progressive tapering of the chronic steroid therapy and the aforementioned antiparasitic treatment cycles, the patient was persistently lymphopenic and parasitemic, and VL relapses occurred several times. The patient also experienced six episodes of urinary tract infections caused by fully susceptible *Escherichia coli*. In three of these cases, the infection progressed to a bloodstream infection requiring hospital admission for intravenous antibiotic treatment. Sixteen months after VL diagnosis, hypogammaglobulinemia was detected; the patient was diagnosed with common variable immunodeficiency and started on intravenous immunoglobulins (IVIG) 0.4 g/kg three times per week.

Twenty months after VL diagnosis, the patient was admitted to the intensive care unit with a recurrence of urinary septic shock and profound pancytopenia. On that occasion, parasitemia was at the highest levels recorded (112,000 parasite equivalents/mL, Fig. 1). In addition to antibiotics for the septic shock, a combination therapy against VL constituted by L-AMB 4 mg/kg for 5 days plus miltefosine 50 mg three times a day for 1 month was carried out, followed by maintenance therapy with L-AMB (4 mg/kg) every month for 6 months. Triweekly IVIG was maintained, while hydroxychloroquine was suspended, and the methylprednisolone dose was further reduced. As VL relapses occurred shortly after denosumab administration (Fig. 1), this drug was suspended indefinitely. The implementation of this therapeutic strategy led to a dramatic drop in the parasitemia and resolution of symptoms. Parasitemia remained consistently negative without any recurrence of symptoms nor pancytopenia at the 3-year follow-up. Osteoporosis was addressed with a reduction in steroid dose and optimization of calcitriol supplementation. The hypogammaglobulinemia also resolved, and the patient was in good clinical condition at the time this paper was finalized.

## DISCUSSION

VL is a major concern in immunocompromised patients, being associated with relapses, resistance to treatment, and reduced survival (2). Our patient suffered from undifferentiated connective tissue disease, for which she was receiving immunosuppressants. At the time of VL presentation, she had just stopped methotrexate, and was receiving oral steroids and hydroxychloroquine; in addition, she was being administered denosumab to treat iatrogenic osteoporosis. During her clinical history, the patient was diagnosed with common variable immunodeficiency, for which she was started on IVIG. Immunosuppression in this patient was a result of both the underlying rheumatological disease and the immunosuppressive drugs; the long-standing *Leishmania* infection could have also contributed to impaired immunity by inducing leukopenia and suppressing T-cell response (6).

In immunocompromised patients, noninvasive direct diagnostic methods, such as molecular testing on peripheral blood, are particularly valuable and qPCR on peripheral blood samples is the most effective technique for monitoring treatment efficacy (7); immunocompromised patients treated for VL should be monitored long-term, possibly for years, to detect possible relapses. In our case, a rise in parasitemia was always accompanied by clinical and laboratory signs of VL. During the disease-free intervals, the patient exhibited a low but constantly detectable parasitemia.

The patient was initially treated for VL with a single drug (firstly L-AMB, total dose 10 mg/kg, secondly miltefosine 50 mg three times a day for 28 days), with VL relapses occurring shortly after treatment completion. These treatment options were suboptimal, considering that the recommended treatment for *Leishmania infantum* VL in immunocompromised individuals is L-AMB 40 mg/kg and that experience with miltefosine is limited (7). Seven months after VL diagnosis, the patient received L-AMB, miltefosine, and allopurinol as the employment of combined treatment has been suggested for VL in immunocompromised patients (8).

Despite the combined antiparasitic treatment, the parasitemia was persistent, with clinical signs of VL during relapses. The subsequent employment of L-AMB 3 mg/kg once a month for 8 months did not improve the clinical course of the parasitic disease. In addition, the patient exhibited lymphopenia and underwent several episodes of sepsis, which suggests a profound impairment of immunity. The ultimate resolution of VL was achieved by an integrated approach that included the following: (i) administration of combined antiparasitic treatment (L-AMB 20 mg/kg total dose plus miltefosine 50 mg three times a day for 1 month) followed by maintenance L-AMB; (ii) tapering of immunosuppression including drastic reduction of oral steroids and suspension of hydroxychloroquine; and (iii) suspension of denosumab.

Denosumab is a monoclonal antibody used to treat osteoporosis by inhibiting the RANKL from binding RANK, thus suppressing bone resorption (3). Besides its role in bone remodeling, denosumab has been associated with an increased risk of infection during the first two years of treatment (9–11). Further, RANKL was reported to inhibit *Leishmania* replication in murine models by inducing fully effector macrophages, with increased killing activity against the parasite (12). In our case, denosumab treatment was started 2 months before the first VL episode, and its suspension was followed by cessation of VL relapses; thus, we hypothesize that this drug may have synergized with the immunosuppressive treatment in inducing *Leishmania* replication and relapsing of VL.

This case highlights the need to consider *Leishmania*-infected immunocompromised patients as complex systems; numerous factors can be implicated in the balance between the parasite, the immune system, and co-medications, and may contribute to the response to treatment.
